# Impact of Pericoronary Microbiota Composition on Course of Recovery after Third Molar Alveotomy

**DOI:** 10.3390/life14050580

**Published:** 2024-04-30

**Authors:** Zrinka Todorić, Milan Milošević, Ivana Mareković, Josip Biočić

**Affiliations:** 1 Department of Clinical Microbiology, Infection Prevention and Control, University Hospital Centre Zagreb, 10000 Zagreb, Croatia; 2 School of Medicine, University of Zagreb, 10000 Zagreb, Croatia; 3 Department for Environmental Health and Occupational and Sports Medicine, Andrija Stampar School of Public Health, 10000 Zagreb, Croatia; 4 Department of Oral and Maxillofacial Surgery, University Hospital Dubrava, 10000 Zagreb, Croatia; 5 School of Dental Medicine, University of Zagreb, 10000 Zagreb, Croatia

**Keywords:** microbiota, third molar alveotomy, recovery

## Abstract

Although the role of microbiota has been investigated in relation to different oral diseases, it is unknown if its composition has any effect on the course of recovery after third molar alveotomy. Our aim was to determine the influence of patient clinical characteristics as well as pericoronary microbiota composition on the course of recovery after a semi-impacted third molar alveotomy. Thirty-six patients were included and samples obtained with paper points, swabs, and tissue samples were analyzed using DNA hybridization and culture methods. Among the 295 organisms detected, the most frequent were *Streptococcus* spp. (22.4%; 66/295) followed by *Fusobacterium* spp. (11.9%; 35/295), and *T. forsythia* (9.1%; 27/295). A comparison of microbiota composition in patients with better and worse recovery did not show significant differences. Worse recovery outcomes were more frequent in patients with a grade 2 self-assessment of oral health (*p* = 0.040) and better recovery courses were observed in patients with a grade 4 self-assessment (*p* = 0.0200). A worse recovery course was statistically significant more frequently in patients with previous oral surgical procedures (*p* = 0.019). Although we demonstrate that worse recovery outcomes were more frequent when certain bacteria were detected, there was no statistically significant difference. Further research is needed to identify microbial profiles specific to the development of worse outcomes after a third molar alveotomy.

## 1. Introduction

A human, as a complex biological system, is not an autonomous organism but has been living in symbiosis with microbiota for millions of years. The composition and diversity of microbiota are fundamental for maintaining the homeostasis of the human organism. When harmful bacteria outnumber commensal or normal microbiota, this is referred to as dysbiosis. Dysbiosis can be caused by many factors, particularly environmental ones, such as diet, the use of antimicrobial agents and food additives, lifestyle habits, hygiene, and host-specific factors including health status and genetic background [1,2]. 

A wide range of microbiota inhabits the human oral cavity. The most prevalent bacterial community is dominated by the six major phyla—Firmicutes, Bacteroidetes, Proteobacteria, Actinobacteria, Spirochaetes, and Fusobacteria. There is still a large proportion of oral bacteria that cannot be cultivated in the laboratory for many reasons, such as a lack of specific nutrients and inadequate temperature and pH [3].

Local changes in microbiota in the oral cavity are thought to contribute to the development of illnesses such as dental caries, periodontal diseases, recurrent aphthous stomatitis, oral tumors, and pericoronitis [4]. Bacteria have a predilection to localize in certain parts of the oral cavity. The subgingival plaque of mandibular third molars has shown remarkable changes in the symptomatic period of pericoronitis, including a significant increase in microbial richness and a convergent trend in microbial composition. It is challenging to maintain proper hygiene in the small pocket surrounded by the soft tissue above the emerging tooth. This creates a favorable habitat for both obligatory and facultative anaerobic bacteria. Moreover, food particles can easily become trapped in this area, encouraging bacterial proliferation and leading to infection of the adjacent soft tissue, commonly known as pericoronitis [5]. After treatment, the subgingival microbiome was altered and largely returned to the state of the asymptomatic period. The number of *Fusobacterium* increased most in subgingival plaque during pericoronitis, according to Huang et al.‘s study [6]. The most prevalent species of *Fusobacterium*, *F. nucleatum*, is a periodontal pathogen linked to a variety of human illnesses, including respiratory tract infections, cardiovascular disease, and gastrointestinal issues. Thus, it is possible that the pathophysiology of acute pericoronitis is directly linked to the increase in *Fusobacterium* [7,8]. According to the literature, the key microorganisms associated with acute pericoronitis are the *Streptococcus anginosus* group and other viridans streptococci, the *Actinomyces* and *Prevotella* genera, *Tannerella forsythia*, *Fusobacterium nucleatum*, *Dialister invisus*, *Treponema denticola*, and *Rothia* spp. The *S. anginosus* group is commonly regarded as part of the normal oral microbiota; however, they are also recognized for their potential to cause severe infections like pulmonary empyema, head and neck abscesses with intracranial extension, and bacteremia [9]. A crucial factor contributing to the pathogenicity of *S. anginosus* group bacteria is their ability to bind soluble fibronectin. This binding capability is closely associated with their capacity to adhere to saliva-coated hydroxyapatite. Following attachment, these bacteria serve as an initiating factor for plaque formation, leading to infections facilitated by extracellular enzymes such as hyaluronidase, DNAse, gelatinase, and collagenase [10]. If left untreated, pericoronitis can result in the extension of the localized infection to adjacent head and neck spaces such as the sublingual, submandibular, parapharyngeal, pterygomandibular, infratemporal, submasseteric, and buccal spaces. Early recognition of these space infections is crucial as delayed treatment may elevate the risk of a life-threatening airway compromise for the patient [6,11,12].

Extraction or alveotomy of the third molar is one of the most frequent procedures in oral surgery. Third molars typically erupt between 17 and 22 years, but often incompletely, and 17–69% of third molars are semi-impacted [13]. Complications occur because of anatomical reasons, such as proximity to the inferior alveolar nerve or the third branch of the fifth cranial nerve, unrestorable caries, caries extending to the pulp, restorative treatment of the second molar because of the position of the semi-impacted molar, odontogenic cysts, and tumors. Because of the irregular position, they often cause the retention of food in the periodontal pocket formed around the crown of the tooth and gingiva. They become ideal media for microbial colonization and cause recurrent episodes of pericoronitis [12,14]. Recurrent episodes of pericoronitis are the main reason for alveotomy. The most common postoperative complications include pain, swelling, and trismus. More severe and rare complications include prolonged bleeding, alveolar osteitis, and damage to nerves and adjacent teeth [15,16,17]. 

Although the role of microbiota has been investigated in relation to different oral diseases, it is unknown if its composition has any effect on the course of recovery after third molar alveotomy. If so, knowledge of the periodontal pocket’s microbiota may be crucial for planning the postoperative recovery process, which may require antibiotic prophylaxis. The results would be significant since the length of recovery has an impact on both quality of life and the return to normal activities. Therefore, the aim of this study was to determine the influence of patient clinical characteristics as well as pericoronary microbiota composition on the course of recovery after a semi-impacted third molar alveotomy. 

## 2. Materials and Methods

This cross-sectional study was performed from March 2019 to October 2022 and included patients ≥ 18 years old without known serious somatic diseases, recruited for an elective alveotomy of semi-impacted mandibular wisdom teeth on a voluntary basis. Inclusion criteria involved recurrent pericoronitis and/or transparency distal to the semi-impacted crown discovered by the panoramic X-ray or CBCT (cone-beam computed tomography system). The latter had to be more than 3 mm in size representing a chronic inflammatory bony socket. All patients had an ASA I score according to The American Society of Anesthesiologists Physical Status Classification System designating otherwise healthy patients. Patients had baseline data recorded including age, gender, comorbidities, allergic reactions, previous pericoronitis episodes, and antimicrobial treatment. Patients with acute pericoronitis upon admission, pregnant women, nursing mothers, people on a specific diet, and people with congenital or acquired pathological conditions and known abuse of opiates, analgesics, or drugs were excluded from the research. Patients who were on antibiotic therapy a month prior were also be excluded. This study was approved by the Ethics Committee of the School of Dental Medicine, University of Zagreb, and informed consent was obtained from the patients included in the study.

All of the selected patients were treated by the same oral surgeon at the Department of Oral and Maxillofacial Surgery, University Hospital Dubrava, Zagreb. Before the surgery, the candidates were asked to rinse their mouths with an oral antiseptic solution containing 0.12% chlorhexidine digluconate (Perio-Aid, Dentaid, Barcelona, Spain) for 30 s. The perioral skin region was treated with a combination of octenidine and phenoxyethanol (Octenisept, Schülke & Mayr GmbH, Norderstedt, Germany). 

After mandibular block and infiltration local anesthesia (Ubistesin forte, 3M Deutschland GmbH, Neuss, Germany), a mucoperiosteal flap was raised and specimens for microbiological analysis were taken. The operation continued with sufficient alveolar bone removal by the rotatory bur in the handpiece and the wisdom tooth was extracted with or without separation of its crown and roots. The flap was repositioned and secured with silk 3-0 sutures. The patients were released with instructions for home oral hygiene, analgesic regimens, and data collection. For the first three days, the patients were contacted by video telephone call to evaluate patient data collection and any possible inconveniences related to the latter. The final clinical control and suture removal was scheduled for the seventh day and the data collected by patients at home were reevaluated by the surgeon.

During the alveotomy, specimens for determining pericoronary microbiota composition were obtained from third molar perialveolar pockets with paper points (size 50). The presence and composition of pericoronary microbiota were determined at the Clinical Department of Clinical Microbiology, Infection Prevention and Control, University Hospital Centre Zagreb, Zagreb, Croatia, by the DNA hybridization method (Micro-Ident^®^ plus 11, Bruker, Berlin, Germany). DNA was isolated from the specimen, amplified, and detected via hybridization and alkaline phosphatase reaction on a membrane strip. This method is capable of detecting 11 of the most common periodontogenic bacterial species—*Aggregatibacter actinomycetemcomitans*, *Porphyromonas gingivalis*, *Prevotella intermedia*, *Tannerella forsythia*, *Treponema denticola*, *Parvimonas micra*, *Fusobacterium nucleatum/periodonticum*, *Campylobacter rectus*, *Eubacterium nodatum*, *Eikenella corrodens*, and *Capnocytophaga* spp. Additionally, swab and tissue samples were obtained during alveotomy procedures and cultivated in aerobic and anaerobic conditions. Grown bacterial isolates were identified using MALDI-TOF MS (MALDI Biotyper Microflex LT/SH, Bruker Daltonics GmbH, Bremen, Germany). Pure isolates were directly spotted onto a MALDI target plate and 1 µL of extraction matrix was added to each spot and allowed to air dry. The inoculated target was placed in the Bruker Biotyper instrument and analyzed using MALDI-TOF to obtain the organism profiles, using the Bruker Bacterial Test Standard for calibration. 

The patients were asked to self-assess their oral health before the alveotomy on a scale from 1 to 4 (1—poor, 2—fair, 3—good, and 4—excellent) as previously described [18]. To evaluate recovery after the procedure, the patients were divided into two groups with regard to better or worse recovery course. A better recovery course referred to the patients in whom the wound healed properly by the seventh postoperative day as determined by clinical examination, while the group with a worse recovery course included patients where disintegrated blood clots and/or alveolitis were present.

In the statistical analysis, we evaluated demographic and clinical characteristics as well as bacterial pathogens. Data for categorical variables are reported as counts and percentages, while quantitative data are reported with medians and interquartile ranges. The Kolmogorov–Smirnov test was used for data normality assessment. Fisher’s exact test was used to analyze differences in categorical data, while the Mann–Whitney U test was used for quantitative data. A comparison of microbiota composition in patients with better and worse outcomes was analyzed using Fisher’s exact test. All *p* values were two-tailed and, if below 0.05, were considered significant. MedCalc^®^ Statistical Software version 22.021 (MedCalc Software Ltd., Ostend, Belgium; https://www.medcalc.org; accessed on 15 February 2024) was used in all statistical calculations.

## 3. Results

During the study period, 36 patients with semi-impacted third molar alveotomy were included. The majority of patients were female (72.2%; 26/36) and the median age of patients was 26.6 years (range 18–44). Demographic characteristics of patients and alveotomy procedure characteristics are shown in Table 1. Additionally, comorbidities found in the study patients were endocrinology diseases (8.3%; 3/36) [polycystic ovary syndrome (n = 1), hyperthyroidism (n = 1), and hypothyroidism (n = 1)], malformations (8.3%; 3/36) [congenital lung defect (n = 1), deviation of the nasal septum (n = 1), arrhythmogenic right ventricular dysplasia (n = 1)], and cardiovascular diseases (5.6%; 2/36). There were no central nervous system, urogenital, liver, or hematological diseases in patients included in this study. Allergic reactions were present in seven patients, but none of them were allergic to anesthetics. These seven patients were allergic to penicillin (n = 4), rabbit hair (n = 1), folacin (n = 1), and pollen, dust, and animal hair (n = 1). The majority of patients (52.8%; 19/36) did not have any antimicrobial treatment before the alveotomy. However, seven patients (19.4%; 7/36) received three courses of antimicrobial treatment. None of the patients had antimicrobial treatment administered 3 months before the alveotomy. 

In 36 patients, a total of 295 organisms were detected by both the DNA hybridization method and cultivation. The distribution of the detected bacterial species is shown in Table 2. The most frequent isolate was *Streptococcus* spp. (22.4%; 66/295), which comprises more than half of all detected isolates. They were followed by *Fusobacterium* spp. (11.9%; 35/295), *T. forsythia* (9.1%; 27/295), *P. micra* (8.8%; 26/295), and *Prevotella* spp. (8.5%; 25/295). 

The most common species among *Streptococcus* spp. Was the *S. anginosus* group (52.0%; 34/66), comprising *S. anginosus* (n = 23), *S. constellatus* (n = 9), and *S. intermedius* (n = 2), followed by the S. mitis group (22.7%; 15/66), *S. mutans* group (16.7%; 11/66), *S. salivarius* group (9.1%, 6/66), and *S. sangiuinis* group (1.5%; 1/66) (Table 3). All the *Fusobacterium* spp. isolates were identified as *Fusobacterium nucelatum/odonticum* and the most common species among *Prevotella* spp. isolates was *P. buccae* (31.6%; 6/19). *Capnocytophaga* spp., also included in a panel of DNA hybridization, was not detected in any of our patients.

A comparison of clinical characteristics and the self-assessment of oral health in patients with better and worse recovery is shown in Table 4. In comparison to patients with a better recovery course, a worse recovery course was more frequently found in female patients (57.1% vs. 30%), older patients (median 27.0 vs. 25.0), patients with a higher body mass index (median 23.9 vs. 22.8 kg/m^2^), and patients with a higher number of pericoronitis episodes before the alveotomy (2 vs. 1), but without statistical significance. Importantly, we demonstrated that worse recovery outcomes were with statistical significance more commonly present in patients with previous oral surgical procedures (95% CI, 1.23 to 49.83, *p* = 0.019). When the self-assessment of oral health was compared, worse recovery outcomes were more frequent in patients with a grade 2 self-assessment (*p* = 0.040) and better recovery outcomes were more frequent in patients with a grade 4 self-assessment (*p* = 0.0200), and both differences were statistically significant (95% CI, 0.10 to 0.98).

A comparison of microbiota composition in patients with better and worse recovery is shown in Table 5. Although *Streptococcus* spp. (23.3% vs. 22.2%), *Fusobacterium* spp. (16.3% vs. 11.1%), *Tannerella forsythia* (11.6 vs. 8.8%), *Treponema denticola* (14.0 vs. 6.3%), *Veilonella* spp. (11.6% vs. 6.7%), and *Lactobacillus* spp. (7.0 vs. 6.3%) were more frequently found in patients with a worse recovery course, there was no statistically significant difference in comparison to the patients with a better recovery course. On the other hand, *P. micra* (9.9% vs. 2.3%), *Prevotella* spp. (8.7% vs. 7.0%), *C. rectus* (5.2% vs. 0.0%), *Eubacterium nodatum* (4.4% vs. 0.0%), *Eikenella corrodens* (4.0 vs. 2.3%), *Actinomyces* spp. (1.6% vs. 0.0%), *P. gingivalis* (1.2% vs. 0.0%), and coagulase-negative staphylococci were more frequently found in patients with a better recovery course, but also without a statistically significant difference in comparison to the patients with a worse recovery course.

## 4. Discussion

Complications associated with third molar alveotomy are estimated to be at about 3–30%. Different studies have assessed patient factors influencing the course of recovery in these patients. Postoperative problems following the extraction of the mandibular third molar were most commonly associated with age and surgical difficulties [19,20,21]. Patients’ ages in our study were comparable to those in other studies (median 25.0 years), and there was no statistically significant age difference between patients who had a worse or better course of recovery. The majority of our patients were females (72.2%; 26/36), which was similar to other studies investigating third molar alveotomy; nevertheless, we did not find evidence to support the hypothesis that gender is a significant predictor of complications and inferior recovery outcomes [18,22,23,24]. The stated comorbidities were those that the patients themselves stated in their portfolios. None of the comorbidities, to the best of our knowledge, influenced the postoperative sequelae after the alveotomy of a wisdom tooth as none of the patients was simultaneously taking analgesics or undergoing corticosteroid therapy. There was no antiplatelet or anticoagulant treatment reported for congenital heart dysplasia nor was there hormone treatment reported for polycystic ovary syndrome. Antibiotic treatment for another purpose was thoroughly investigated for exclusion. Furthermore, all the diseases and states mentioned were under the control of a pertaining specialist and we could not find a reason to exclude them from this study. We demonstrated that worse recovery outcomes were statistically significant more frequently in patients with previous oral surgical procedures (*p* = 0.019) as well as in patients who had a grade 4 self-assessment of oral health (*p* = 0.0200) before the alveotomy. Previous oral surgery procedures and the self-assessment of oral health grade might influence to a certain extent the recovery situation after third molar surgery. History of oral surgery treatment makes it easier for experienced patients to be aware and better prepare for the upcoming recovery period. Good oral hygiene needs to be continued after the procedure and the quality of it should meet high standards. Postoperative pain, swelling, and trimus make it difficult for a patient to perform oral hygiene at the mentioned levels. The questionnaire for the self-assessment of the quality of oral health provided insight into the respondents’ satisfaction with their own oral health and how it affects their self-confidence, daily life, work, interpersonal relationships, and general satisfaction with their own life. It has been shown that oral health is important not only for health and aesthetic reasons but it also has great sociological significance as it contributes to overall satisfaction with one’s life.

Recently, oral microbiota and its changes were investigated and are thought to contribute to the development of dental caries, periodontal diseases, recurrent aphthous stomatitis, oral tumors, and pericoronitis [25,26,27]. Our study investigated the influence of pericoronary microbiota on the recovery course of patients with a semi-impacted third molar alveotomy after having clinical and radiological evidence of recurrent pericoronitis in their medical history. Similar to other studies including patients with pericoronitis, streptococci were the most frequently isolated bacteria from perialveolar pockets in our patients [28]. Viridans streptococci are divided into six major groups: (1) *mitis* group, (2) *salivarius* group, (3) *anginosus* group, (4), *mutans* group, (5) *sanguinis* group, and (6) *bovis* group [29,30]. The recovery rate of the *S. anginosus* group bacterial isolates is heavily influenced by the specific selective media employed for the initial culture of the samples [31,32]. In our study, the utilization of Columbia agar guaranteed a high retrieval rate of streptococci, which could explain the comparatively higher recovery rates of *S. anginosus* group bacteria in contrast to other studies. It is important to note that some of the previous studies, which primarily concentrated on cultivating obligate anaerobic bacteria and did not employ media for streptococci retrieval, should not undermine the significance of the *S. anginosus* group. In a study by Sencimen et al., streptococci were not detected, but they were not included in the microbiological work-up [32]. A study by Huang et al., in which 16S rRNA sequencing was used, found that when pericoronitis of the third molar occurred, *Streptococcus* spp. increased largely in the saliva, while the amount of *Fusobacterium* spp. and *Neisseria* spp. increased significantly in subgingival plaque [6]. The most common species among *Streptococcus* spp. in our patients was the *S. milleri* group (52.0%; 34/66). In previous studies, it was concluded that the *S. anginosus* group bacteria, well-known for their ability to cause suppurative infections, are most likely involved in the pathogenesis of acute severe pericoronitis of the lower third molar [33]. *F. nucleatum*, the second most frequent bacterial isolate in our study, is known to be a bridging species that adheres to both early commensal streptococci as well as periopathogens such as *Porphyromonas gingivalis*, *Treponema denticola*, and *Tannerella forsythia*, which were also found in our study [34,35,36,37,38].

The influence of oral microbiota on the recovery course after a third molar alveotomy has not been investigated previously. Although, in our study, a worse recovery course was more frequent when bacterial species such as *Streptococcus* spp., *Fusobacterium* spp., *T. forsythia*, *T. denticola*, *Veilonella* spp., and *Lactobacillus* spp. were detected, there was no statistically significant difference in comparison to the patients with a better recovery course. Studies investigating the role of microbiota in postoperative recovery were, however, conducted in patients after gastrointestinal surgery procedures. It is still unclear why some patients develop complications and others do not, even though cases seem to share common clinical features. In the last decade, involvement of the gut microbiota composition in the development of postoperative complications has been suggested [39,40,41]. The relative abundance of enterococci in the anastomotic tissue of rats, for instance, increased 500-fold, according to research by Shogan et al. [42]. As a result of tissue degradation, which may be the cause of anastomotic leakage, *Enterococcus faecalis* can help break down collagen and activate the enzyme tissue matrix metalloprotease-9 (MMP9) in the intestinal tissue of the host [43].

Difficult postoperative recovery has a multifactorial etiology, but the microbiological composition of the oral flora and the pericoronary area certainly play an important role. Today, there is no definitive position regarding antibiotic prophylaxis in the alveotomy of mandibular wisdom teeth. The incidence of infection after alveotomy of the lower wisdom teeth ranges between approximately 0.8 and 4.2% [44]. Furthermore, difficult and painful alveolar healing is frequently closely related to infectious factors. One of the goals of our research was to determine whether there is a connection between the composition of the microbiota status and troublesome postoperative recovery and wound healing. Our research showed a higher incidence of certain pathogens, but it was not statistically significant. Consequently, the controversy around the requirement for antimicrobial prophylaxis during this procedure could not be resolved by the information about variation in microbiota composition. In our study, BMI was not significantly different between the patients with better and worse recovery (*p* = 0.780). BMI is an anthropometric measure that quantifies relatively well the amount of body fat. It has long been known that elevated values have a clear correlation with a number of diseases, such as diabetes, hypertension, and obesity, just as low values can indicate anorexia. During a series of studies, it has been proven that increased BMI is correlated with lower periodontal oral health and poorer oral hygiene and diseases such as caries, pericoronitis, and tooth loss. In contrast, a normal BMI is associated with better periodontal oral health. Of course, sociological factors, such as lifestyle, dietary habits, and oral health awareness, also contribute to the correlation between BMI and oral health [45,46,47]. However, worse recovery outcomes were more frequent in patients with a grade 2 self-assessment (*p* = 0.040) and better recovery outcomes were more frequent in patients with a grade 4 self-assessment (*p* = 0.0200), and both differences were statistically significant. The self-assessment grade before the procedure may be an additional factor in deciding whether a patient requires antimicrobial prophylaxis or not.

There is no clear guidance on the administration of antimicrobial prophylaxis. However, a single pre-operative dose of antibiotics seems to be sufficient for the majority of impacted wisdom teeth [48]. Nonetheless, the adverse effects of antimicrobial agents which have been stated to occur in 6% to 7% of patients and the potential risks of antimicrobial resistance must be considered [49]. A single 2 g dose of amoxicillin still remains the first choice in such instances. A combination with clavulanic acid might be an option where beta-lactamase-producing microbes are present. A total of 60 mg of clindamycin is reserved for candidates allergic to penicillin; however, clinicians must bear in mind that up to 24% of *Streptococcus pyogenes* were already resistant to it by 2018 [50]. Recent studies are showing that the susceptibility of viridans streptococci, the most common isolates in our study, is declining. In a study by Singh et al., susceptibility rates of >90% for many clinically relevant antibiotics for viridans streptococci including ceftriaxone, meropenem, levofloxacin, and vancomycin were observed. Isolates identified as *S. mitis* had notably lower susceptibility rates to penicillins and macrolides [50]. Kim et al. obtained 635 viridans streptocci isolates from dental plaques and identified 154 *S. oralis*, 136 *S. mitis*, 129 *S. anginosus*, 123 *S. sanguinis*, 33 *S. salivarius*, 27 *S. mutans*, 22 *S. gordonii*, and 11 *S. constellatus* species. Almost all of the isolates were sensitive to amoxicillin (99.6%) and cefotaxime (99.4%) and all were sensitive to vancomycin; however, some *S. constellatus* (9.1%) and *S. oralis* (0.6%) isolates were resistant to amoxicillin [51]. This difference in susceptibility suggests that it may be beneficial to routinely define the species of viridans streptococci isolates in the clinical microbiology laboratory to better facilitate antibiotic selection.

Our study has two limitations. The first limitation is the fact that we did not use certain sequencing methods like next-generation sequencing (NGS) or whole-genome sequencing (WGS) for the detection of the oral microbiome [52,53,54,55,56]. Cases are described where organisms causing infection after third molar extraction could only be identified by molecular techniques such as 16S RNA gene analysis or NGS [57] Nonetheless, our objective was to identify pericoronary microbiota using techniques that are practical in everyday work, making them simple to apply. Sequencing methods are still technically demanding, expensive, and time-consuming, requiring bioinformatics capabilities. Considerable work still has to be performed to streamline the WGS workflow, especially to speed up the turnaround times for library preparation and WGS platform runs while also cutting expenses even further. Furthermore, user-friendly analytical software and automated platforms for data analysis need to be created [58]. 

The second limitation is that we did not test antimicrobial susceptibility to antimicrobial agents of the bacterial isolates recovered in culture, especially viridans streptococci. These data would be important for determining if amoxicillin is still the optimal choice for antimicrobial prophylaxis. Penicillin susceptibility rates obtained by Croatian national surveillance in 2022 for *Prevotella* spp. and *F. necrophorum*, which we also discovered in our study, were 43.0% and 78.0%, respectively [56,59].

## 5. Conclusions

Overall, we found some significant findings in this study. We found that grade 2 (fair) oral health self-assessments by patients and prior oral surgical procedures are two clinical characteristics that might predict a poorer recovery outcome. When specific bacteria were found, we showed that worse recovery outcomes were more common, although there was no statistically significant difference when compared to individuals who had a better course of recovery. Therefore, our findings did not solve the question of antimicrobial prophylaxis—although the self-assessment of oral health as an additional factor that can help in deciding whether prophylaxis is needed for a particular patient was identified. In order to prevent postoperative complications and identify high-risk patients, more research is required to determine the microbial profile specific to the development of worse outcomes and complications following a third molar alveotomy.

## Figures and Tables

**Table 1:** Demographic and clinical characteristics of patients with alveotomy procedure (n = 36).

Characteristic	Median	Interquartile Range
Age (y)	25.0	23.0–31.0
Body mass index (kg/m^2^)	22.9	21.4–25.3
Number of pericoronitis episodes before alveotomy	2.0	1.0–5.0
Duration of alveotomy (min)	13.0	10.0–16.8
Initial mouth opening (mm)	50.0	45.0–53.0
First analgetic administration (hours after procedure)	1.0	1.0–2.0

**Table 2:** Organisms detected in perialveolar pockets during third molar alveotomy (n = 295).

Bacterial Species	n	%
*Streptococcus* spp.	66	22.4
*Fusobacterium* spp.	35	11.9
*Tannerella forsythia*	27	9.1
*Parvimonas micra*	26	8.8
*Prevotella* spp.	25	8.5
*Treponema denticola*	22	7.5
*Veilonella* spp.	22	7.5
*Lactobacillus* spp.	19	6.4
*Campylobacter rectus*	13	4.4
*Eubacterium nodatum*	11	3.7
*Eikenella corrodens*	11	3.7
*Bifidobacterium* spp.	6	2.0
*Actinomyces* spp.	4	1.4
*Porphyromonas gingivalis*	3	1.0
*Aggregatibacter* spp.	3	1.0
*Coagulase-negative staphylococci*	2	0.7
Total	295	

**Table 3:** Distribution of different species among streptococcal isolates (n = 66).

Bacterial Species	n	%
*Streptococcus anginosus* group	34	51.5
*S. anginosus*	23	34.8
*S. constellatus*	9	13.6
*S. intermedius*	2	3.0
*Streptococcus mitis* group	15	22.7
*S. oralis*	8	12.1
*S. mitis*	2	3.0
*S. parasanguinis*	2	3.0
*S. massiliensis*	1	1.5
*S. cristatus*	1	1.5
*Streptococcus mutans* group	11	16.7
*S. mutans*	8	12.1
*S. sobrinus*	3	4.5
*Streptococcus salivarius* group	6	9.1
*S. vestibularis*	4	6.1
*S. salivarius*	2	3.0
*Streptococcus sanguinis* group	1	1.5
*S. sanguinis*	1	1.5
Total	66	

**Table 4:** Comparison of clinical characteristics and self-assessed oral health in patients with better and worse recovery.

	Better Recovery Course (n = 29)	Worse Recovery Course (n = 7)	*p* Value (Two-Tailed)	OR (95% CI) ***
Gender: n (%)				2.35 (0.42–13.18)
Female gender: n (%)	22 (75.9)	4 (57.1)	0.370	
Male gender: n (%)	7 (24.1)	3 (42.9)		
Age (y): median (* IQR)	25.0 (22.5–31.0)	27.0 (24.0–35.0)	0.434	1.04 (0.93–1.15)
Body mass index (kg/m^2^): median (IQR) Previous oral surgery procedures: median (IQR)	22.8 (21.3–25.5) 0 (0.0–0.5)	23.9 (21.9–24.9) 1 (0.0–1.0)	0.780 0.019	0.99 (0.77–1.29) 7.85 (1.23–49.83)
Number of pericoronitis episodes before alveotomy: median (IQR)	2 (1.0–5.0)	1 (1.0–5.0)	0.731	
** Oral health assessment: n (%)				0.96 (0.81–1.14)
Grade 1	1 (3.4)	0 (0.0)	0.625	
Grade 2	3 (10.3)	3 (42.9)	0.040	
Grade 3	11 (37.9)	4 (57.1)	0.362	
Grade 4	14 (48.3)	0 (0.0)	0.020	

IQR * = interquartile range; ** grading of oral health before alveotomy: 1—poor, 2—fair, 3—good, and 4—excellent; *** confidence interval.

**Table 5:** Comparison of microbiota composition in patients with better and worse recovery.

	Better Recovery Course (n = 252)	Worse Recovery Course (n = 43)	*p*
**Bacterial isolates**			
*Streptococcus* spp. (n = 66)	56 (22.2)	10 (23.3)	0.873
*Fusobacterium* spp. (n = 35)	28 (11.1)	7 (16.3)	0.330
*Tannerella forsythia* (n = 27)	22 (8.7)	5 (11.6)	0.542
*Parvimonas micra* (n = 26)	25 (9.9)	1 (2.3)	0.104
*Prevotella* spp. (n = 25)	22 (8.7)	3 (7.0)	0.712
*Treponema denticola* (n = 22)	16 (6.3)	6 (14.0)	0.075
*Veilonella* spp. (n = 22)	17 (6.7)	5 (11.6)	0.257
*Lactobacillus* spp. (n = 19)	16 (6.3)	3 (7.0)	0.862
*Campylobacter rectus* (n = 13)	13 (5.2)	0 (0.0)	0.127
*Eubacterium nodatum* (n = 11)	11 (4.4)	0 (0.0)	0.162
*Eikenella corrodens* (n = 11)	10 (4.0)	1 (2.3)	0.588
*Bifidobacterium* spp. (n = 6)	5 (2.0)	1 (2.3)	0.897
*Actinomyces* spp. (n = 4)	4 (1.6)	0 (0.0)	0.404
*Porphyromonas gingivalis* (n = 3)	3 (1.2)	0 (0.0)	0.471
*Aggregatibacter* spp. (n = 3)	2 (0.8)	1 (2.3)	0.366
Coagulase-negative staphylococci (n = 2)	2 (0.8)	0 (0.0)	0.556

## Data Availability

The raw data supporting the conclusions of this article will be made available by the authors on request.
